# Supplementary material for: Gentamicin Population Pharmacokinetics in Pediatric Patients—A Prospective Study with Data Analysis Using the saemix Package in R
Source: Pharmaceutics. 2021 Oct 1;13(10):1596. doi: 10.3390/pharmaceutics13101596 (PMC8541459; doi:10.3390/pharmaceutics13101596)
Supplement: Supplementary file 1 [file pharmaceutics-13-01596-s001.zip › pharmaceutics-1370190-supplementary.pdf]

pharmaceutics

MDPI

Supplementary Material: Gentamicin Population Pharmacokinetics in Pediatric Patients—A Prospective Study with Data Analysis Using the saemix Package in R

Paolo Paioni *, Vera F. Jäggi, Romy Tilen, Michelle Seiler, Philipp Baumann, Dominic S. Bräm, Carole Jetzer, Robin T. U. Haid, Aljoscha N. Goetschi, Roland Goers, Daniel Müller, Diana Coman Schmid, Henriette E. Meyer zu Schwabedissen, Bernd Rinn, Christoph Berger * and Stefanie D. Krämer *

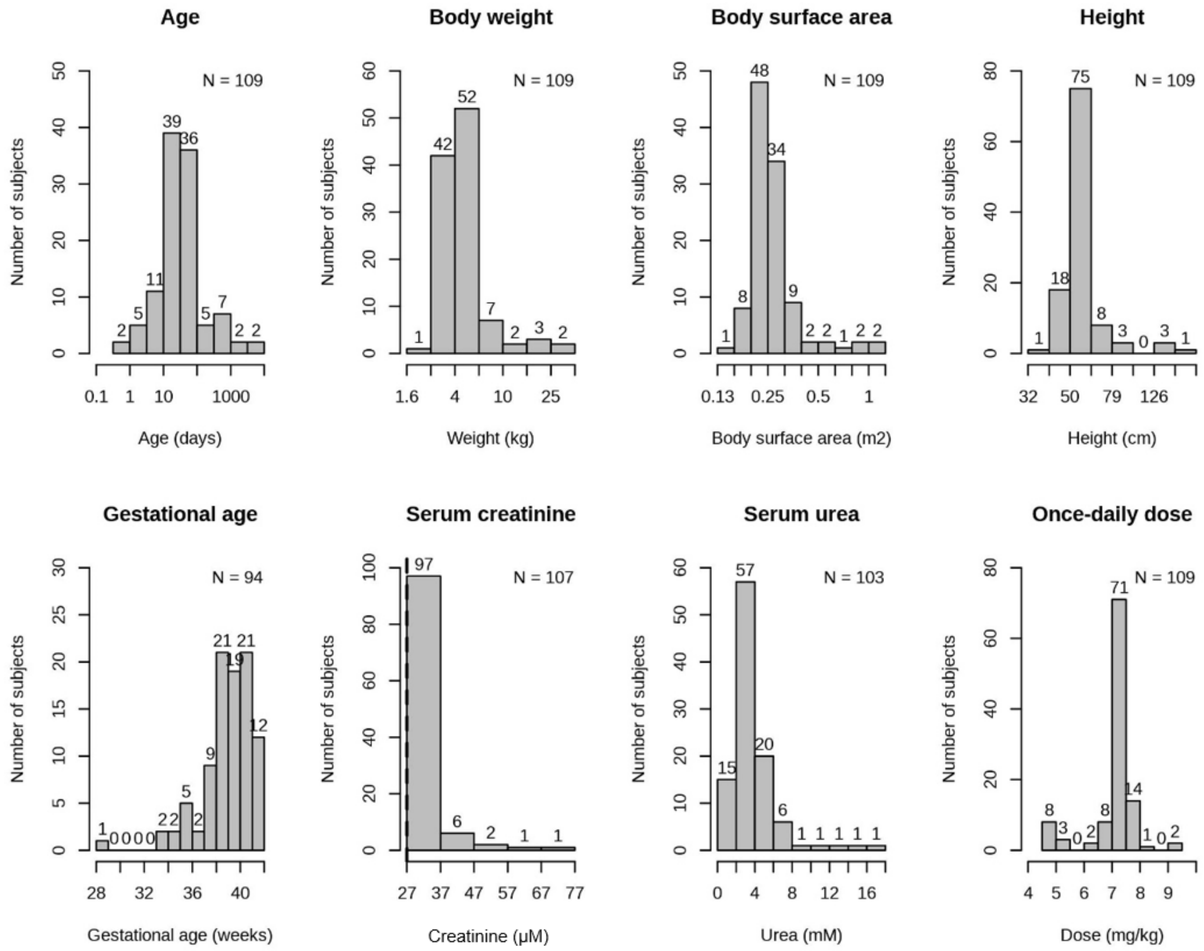

Figure S1. Distribution of the characteristics of the 109 patients (67 male, 42 female). The high bar at 27 μM serum creatinine contains all subjects with < 27 μM serum creatinine (two values, 19 and 25 μM were included in the "<27 μM" group). Mean, median, range and standard deviations are shown in Table 1. Body surface area was calculated from height and weight as 1 (m^2) × (Height (cm) × Weight (kg) / 3600 (cm × kg))$^{0.5}$. The x axes for age, weight, body surface area and height are in logarithmic scale.

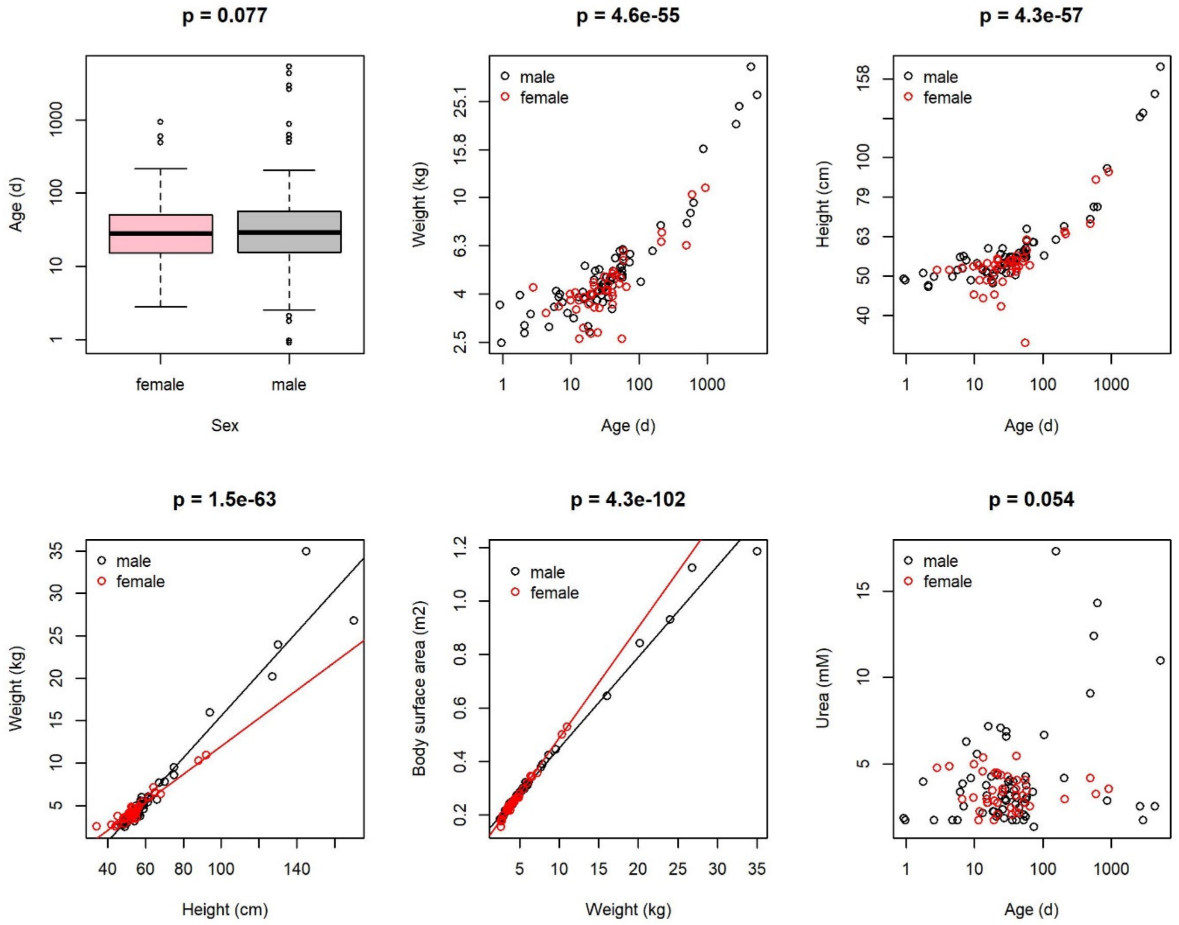

Figure S2. Comparisons of patient characteristics. *p* Values of two-sided homoscedastic t-test (comparing age of female and male patients) and linear correlations, respectively, are shown on top of the graphs (all tests, linear scale). Black symbols, males; red symbols, females. Surface area was calculated from height and body weight as shown in Figure S1. Some axes are in logarithmic scale.

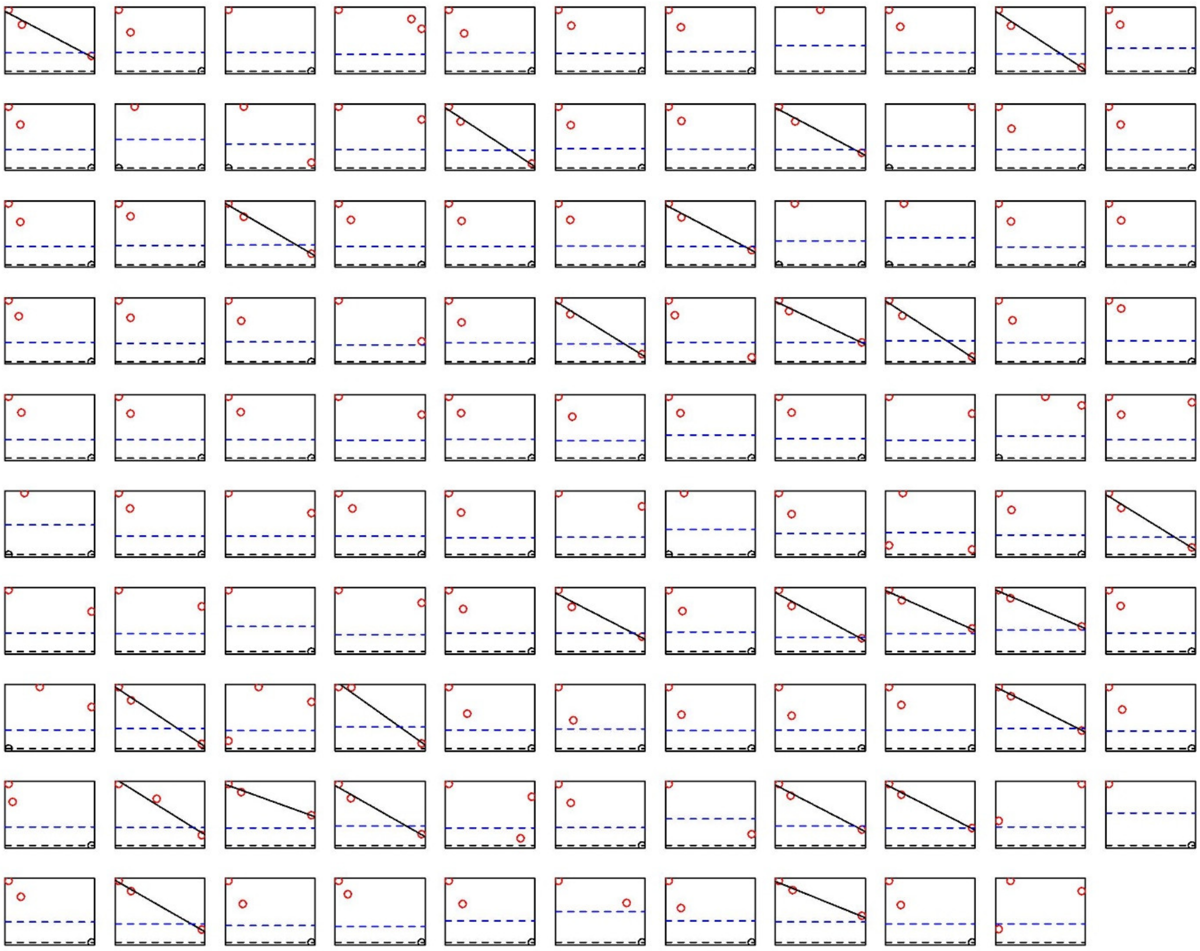

Figure S3. Natural logarithm of gentamicin plasma concentrations of each subject (vertical axis) plotted against time (horizontal axis). Red symbols, concentrations above LOQ (0.3 mg/l); black symbols, concentrations below LOQ (indicated at LOQ, horizontal broken black line); horizontal broken blue line, recommended maximal trough level (1 mg/l). Solid line, linear regression line if more than 2 concentrations above the LOQ were available within one dosing interval (N=24). The general overestimation of the second ln(C) by the linear regression (except two plots) indicates that the data follow a two-compartment rather than one-compartment model. For a one-compartment model, the ln(C(t)) of one dosing interval would in theory follow a straight line in the plots (C(t) as an exponential function of time) and, therefore, randomly scatter around the regression line. The observed distribution of the ln(C(t)) along the linear regression line indicates that C(t) rather follow a bi-enponential function of time, *i.e.*, a two-comapartment model. Axes are individually scaled to the respective data. Note that plots may contain 3 concentrations > LOQ from 2 different dosing intervals and, therefore, contain no regression line.

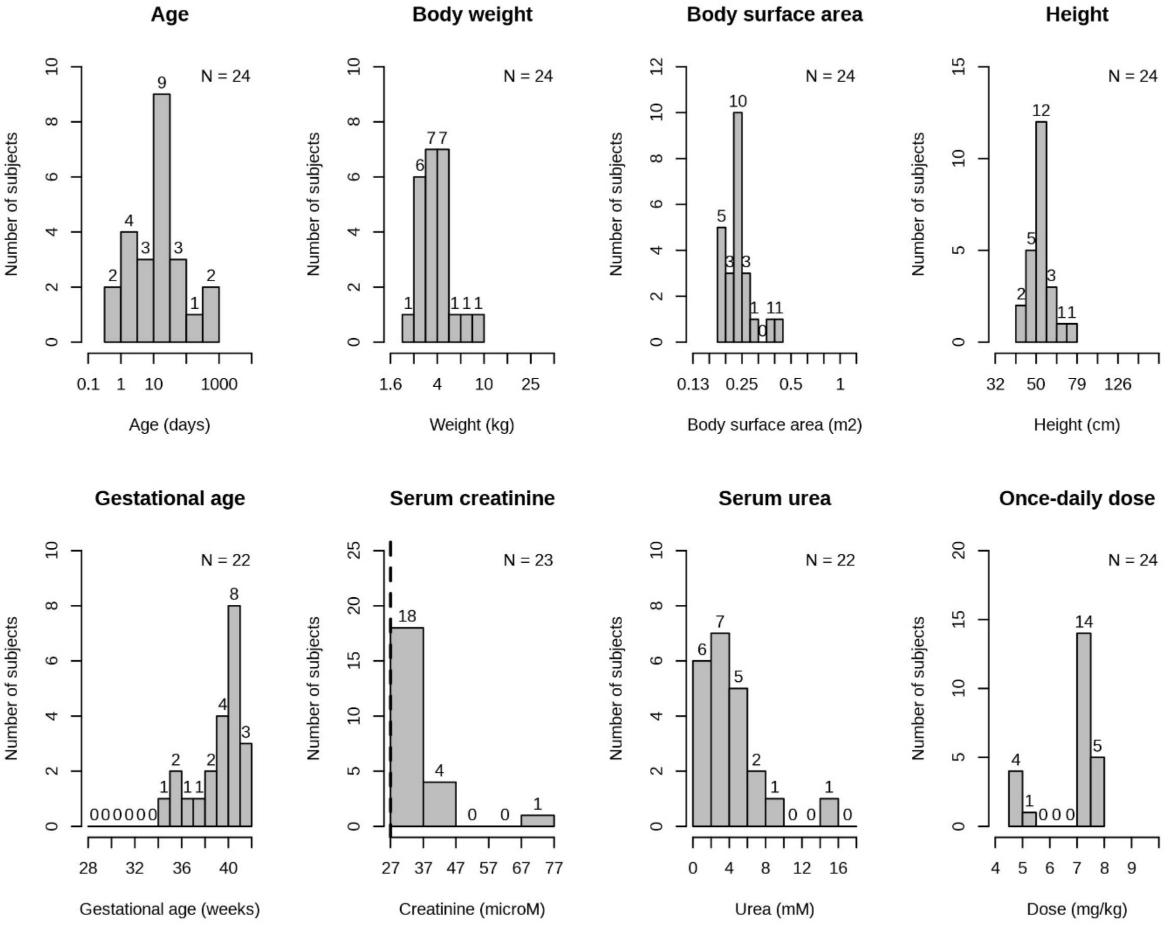

Figure S4. Characteristics of the 24 patients with 3 gentamicin plasma concentrations above the LOQ (0.3 mg/l) within one dosing interval. 15 male, 9 female.

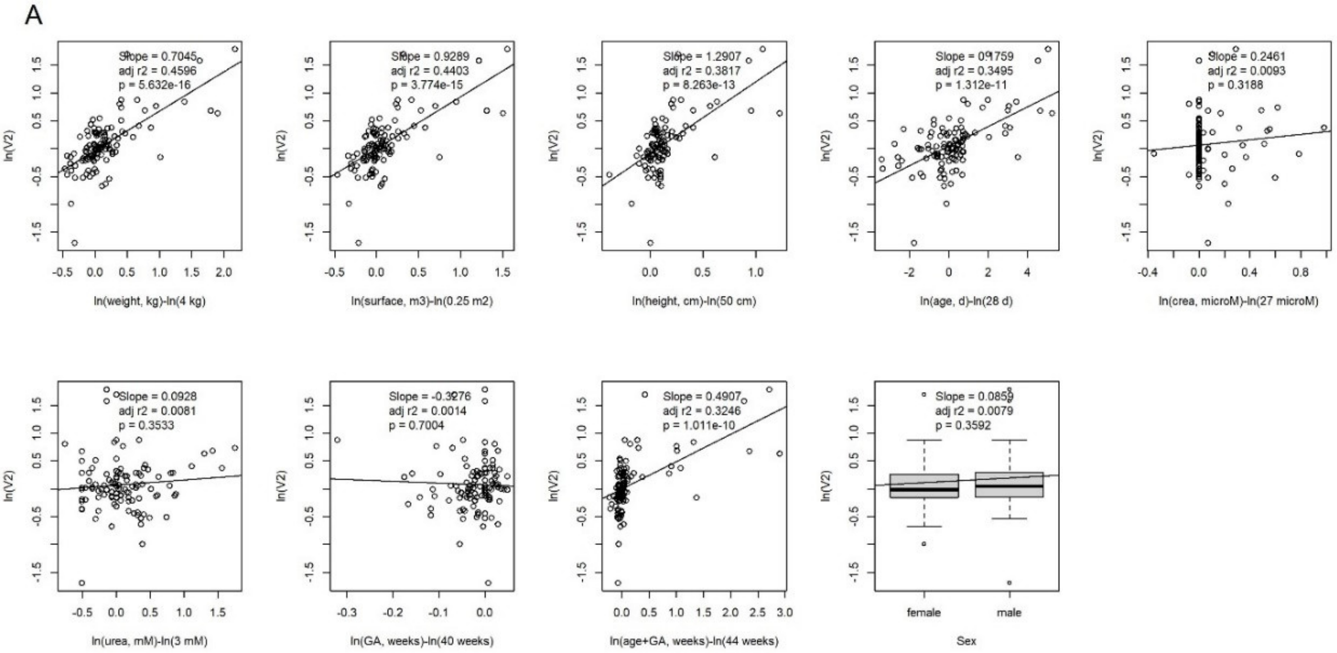

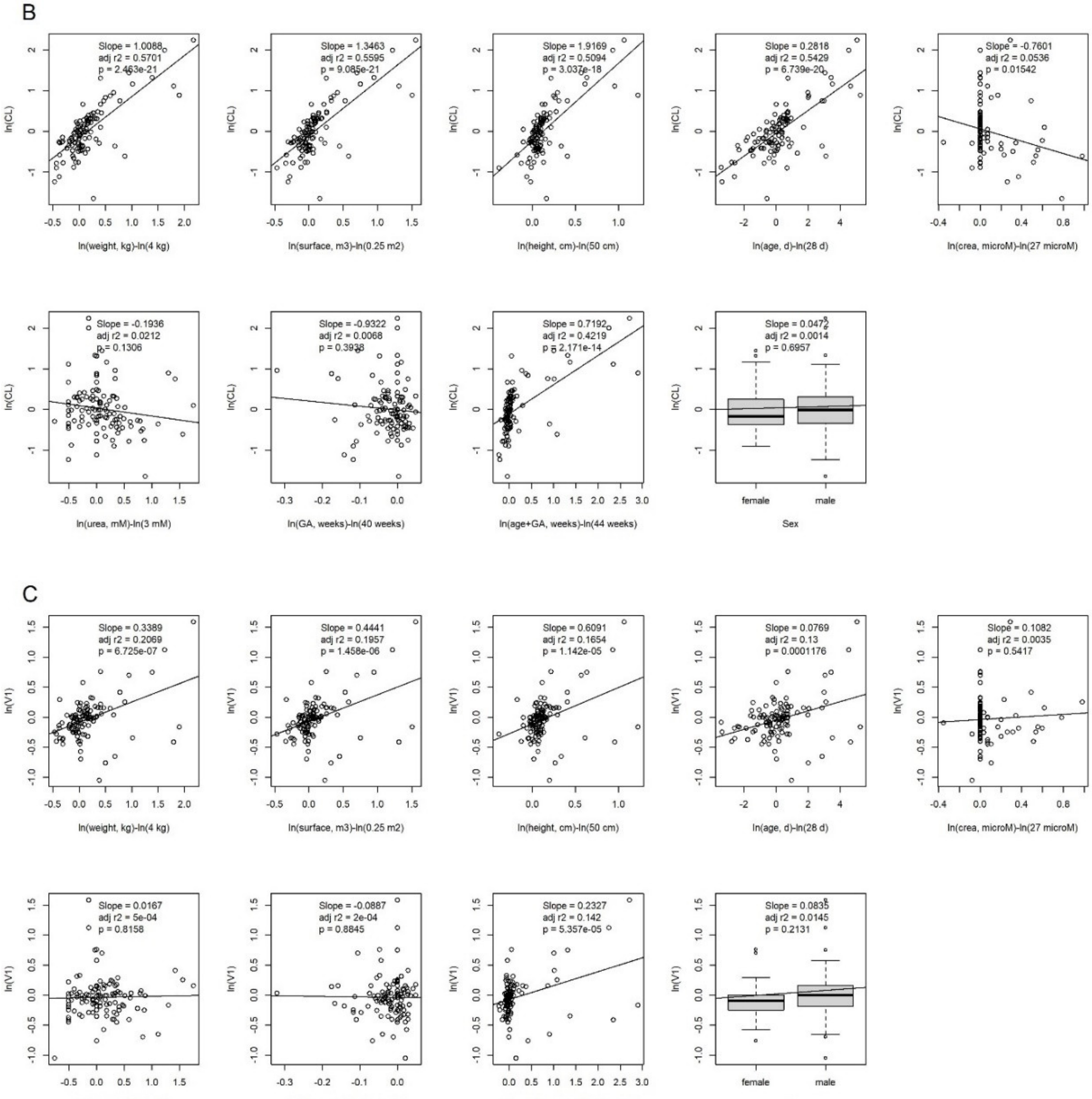

Figure S5. Comparison between patient characteristics and the random effects in the absence of covariates in the model. **A**) For ln(V_2', l), **B**) for ln(CL, l/d) and **C**) for ln(V_1, l). Slope, adjusted r^2 and p of the linear regression are indicated in the individual plots. The random structure was diagonal, without random effects for ln(λ_1).

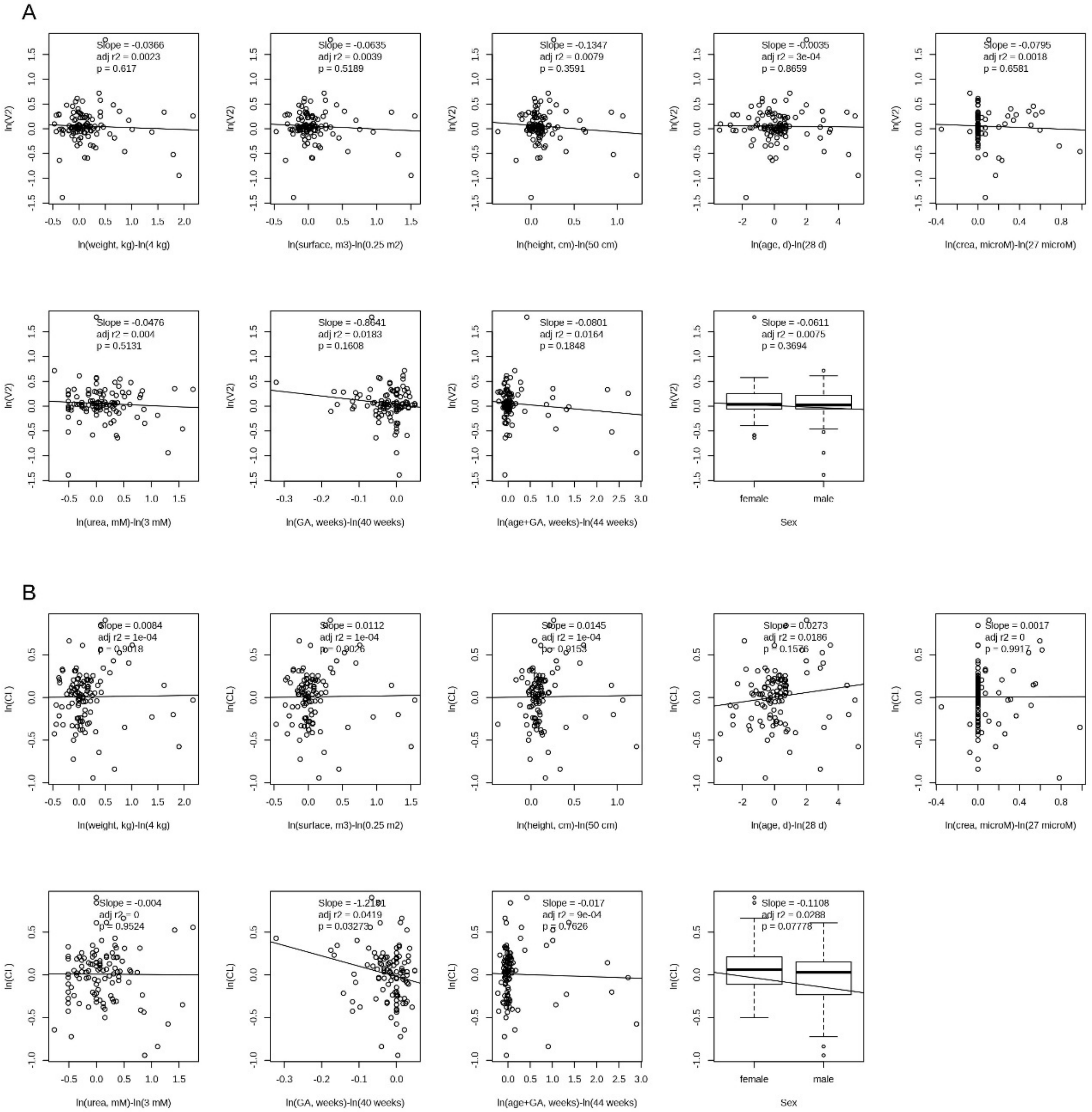
A
Slope = -0.0366
adj r2 = 0.0023
p = 0.617
ln(weight, kg)-ln(4 kg)
Slope = -0.0635
adj r2 = 0.0039
p = 0.5189
ln(surface, m3)-ln(0.25 m2)
Slope = -0.1347
adj r2 = 0.0079
p = 0.3591
ln(height, cm)-ln(50 cm)
Slope = -0.0035
adj r2 = 3e-04
p = 0.8659
ln(age, d)-ln(28 d)
Slope = -0.0795
adj r2 = 0.0018
p = 0.6581
ln(crea, microM)-ln(27 microM)
Slope = -0.0476
adj r2 = 0.004
p = 0.5131
ln(urea, mM)-ln(3 mM)
Slope = -0.8641
adj r2 = 0.0183
p = 0.1608
ln(GA, weeks)-ln(40 weeks)
Slope = -0.0801
adj r2 = 0.0164
p = 0.1848
ln(age+GA, weeks)-ln(44 weeks)
Slope = -0.0611
adj r2 = 0.0075
p = 0.3694
female
male
Sex
ln(V2)
B
Slope = 0.0084
adj r2 = 1e-04
Slope = 0.0112
adj r2 = 1e-04
Slope = 0.0145
adj r2 = 1e-04
Slope = 0.0273
adj r2 = 0.0186
Slope = 0.0017
adj r2 = 0
p = 0.9917
Slope = -0.004
p = 0.9524
adj r2 = 0.0419
p = 0.03273
Slope = -0.017
adj r2 = 9e-04
Slope = -0.1108
adj r2 = 0.0288
p = 0.07778
ln(CL)

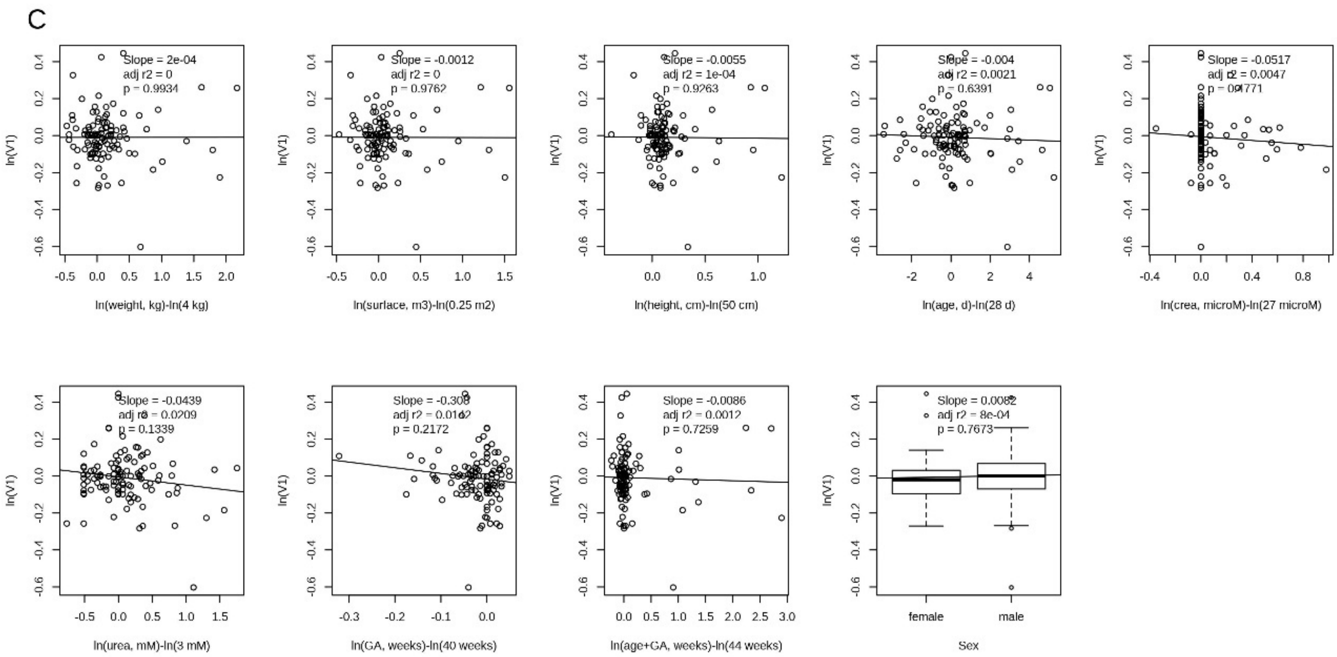

Figure S6. Comparison between patient characteristics and the random effects in the final model. **A**) For ln(V_2'), **B**) for ln(CL) and **C**) for ln(V_1). Included covariates were ln(body weight) for all 3 parameters, and ln(creatinie concentration) and ln(urea concentration) for ln(CL). See Figure S5 for futher details.

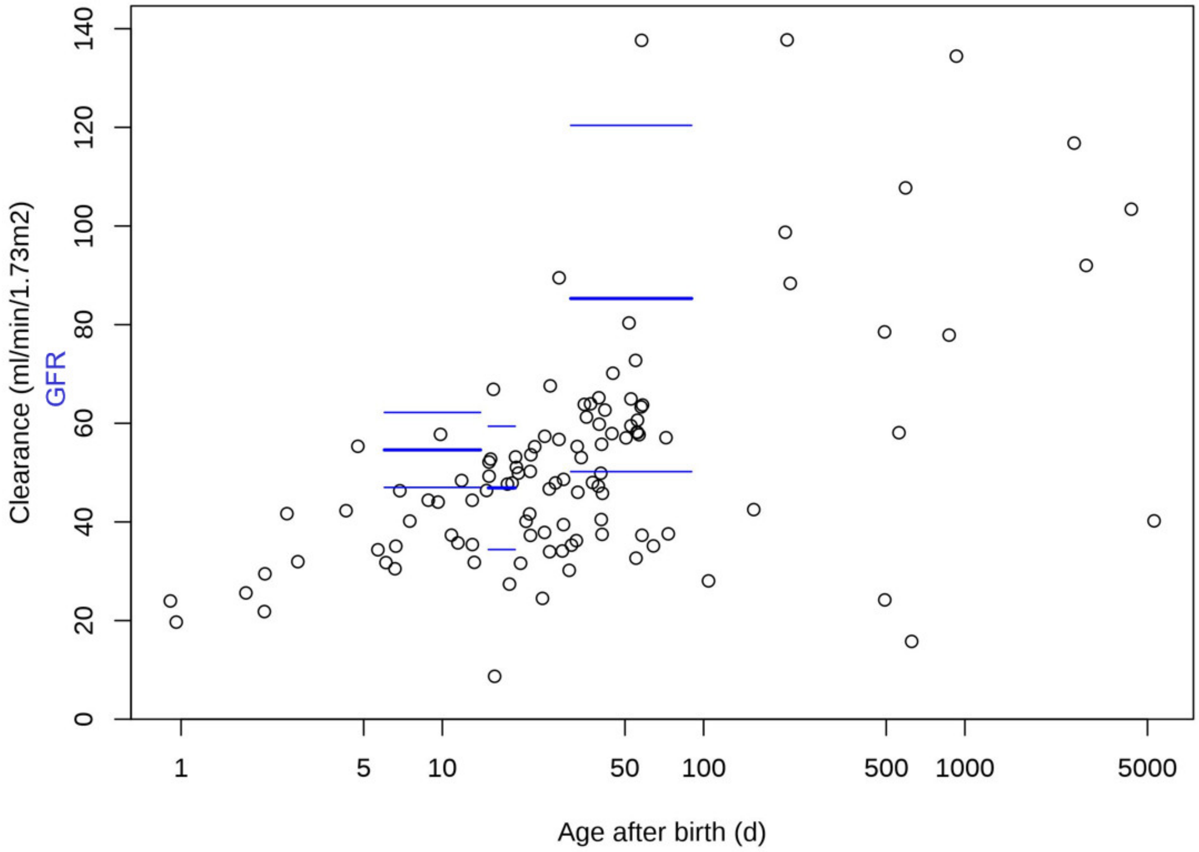

Figure S7. Calculated gentamicin CL (subject level, normalized to 1.73 m² body surface area) compared to age and published GFR (mean values, thick blue lines; SD, thin blue lines; from Schwartz and Forth, 2007 [1]).

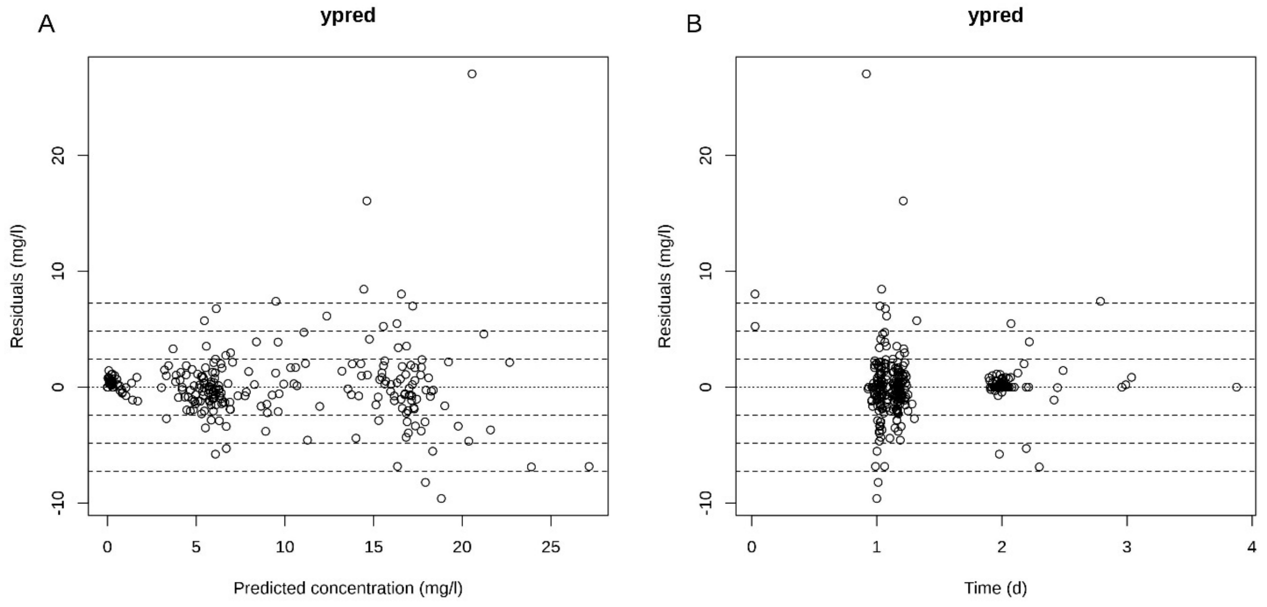

Figure S8. Residuals at the population level of the final model. Residuals plotted against **A**) the simulated values and **B**) time. Dotted line, 0; broken lines, 1, 2 and 3 SD from 0.

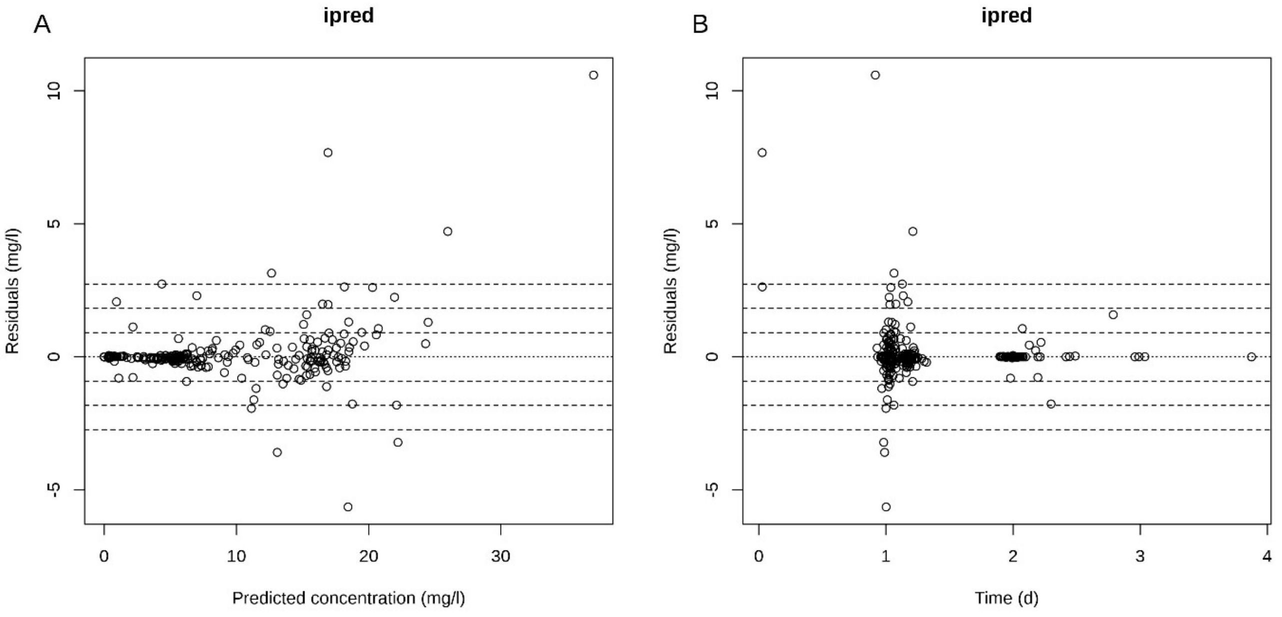

Figure S9. Residuals at the individual level of the final model. Residuals plotted against **A**) the simulated values and **B**) time. Dotted line, 0; broken lines, 1, 2 and 3 SD from 0.

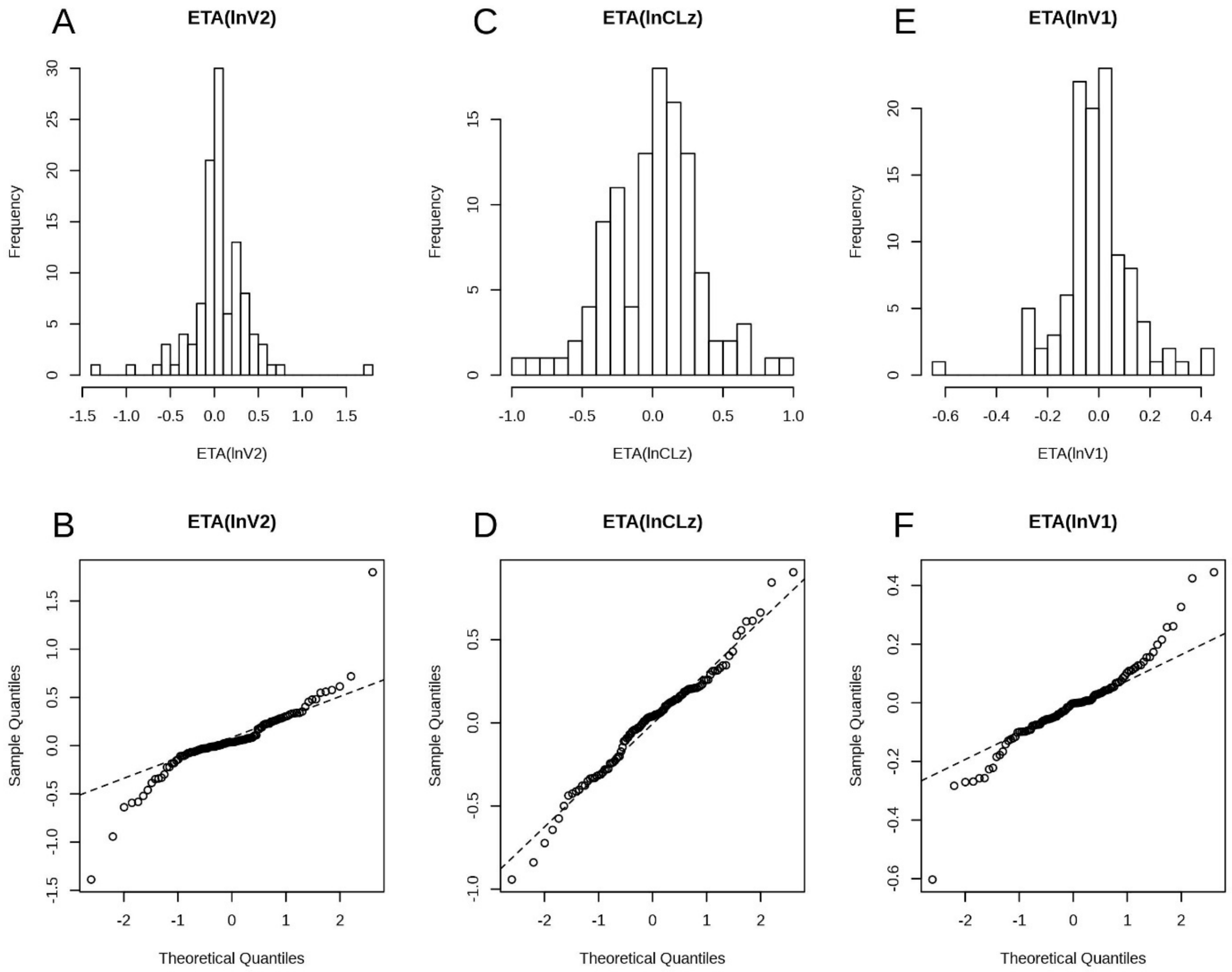

Figure S10. Histograms (**A**,**C**,**E**) and Q-Q plots (**B**,**D**,**F**) of the random effects. **A,B**) ln(V_2'), **C,D**) ln(CL), **E,F**) ln(V_1). Q-Q plot x-axis, theoretical quantiles of a normal distribution with mean = 0 and standard deviation = 1. Broken lines, theoretical normal quantile-quantile plot which passes through the first and third quartiles (*qqline* function of the R package saemix). No random effects were included in the final model for ln(λ_1).

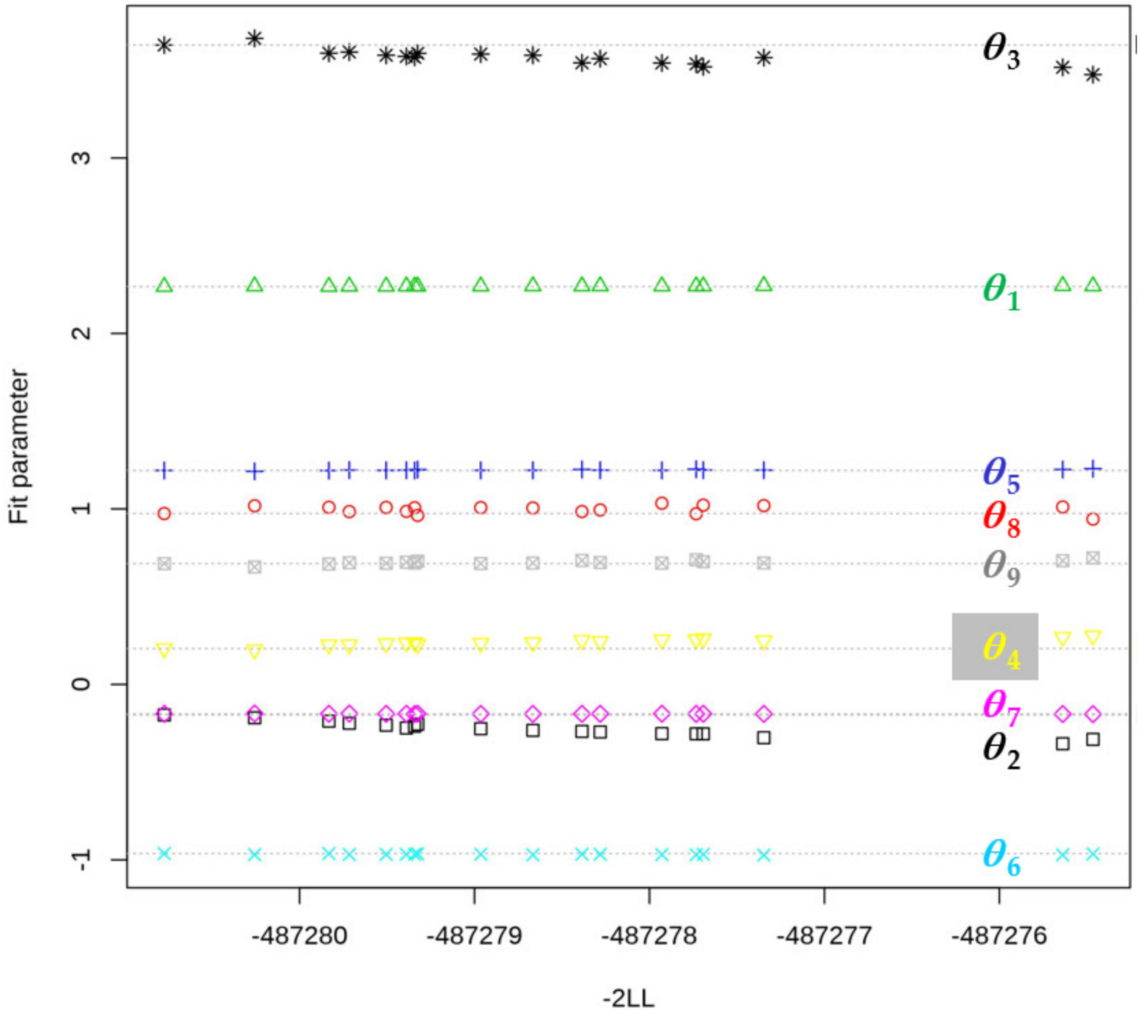

Figure S11. Fit parameters from a multistart analysis with 18 starts with 18 different start parameter sets with randomly chosen paramesters within the ranges provided in Table S1. θ_1, ln(CL) intercept; θ_2, ln(V_2′) intercept; θ_3, ln(λ_1) intercept; θ_4, ln(V_1) intercept; θ_5, factor for ln(CL) covariate [ln(weight)-ln(4kg)]; θ_6, factor for ln(CL) covariate [ln(creatinine)-ln(27μM)]; θ_7, factor for ln(CL) covariate [ln(urea)-ln(3mM)]; θ_8, factor for ln(V_2′) covariate [ln(weight)-ln(4kg)]; θ_9, factor for ln(V_1) covariate [ln(weight)-ln(4kg)]; see Table 3 in manuscript. Best fits are at lowest -2LL. Dotted horizontal lines, fit parameters at lowest -2LL.

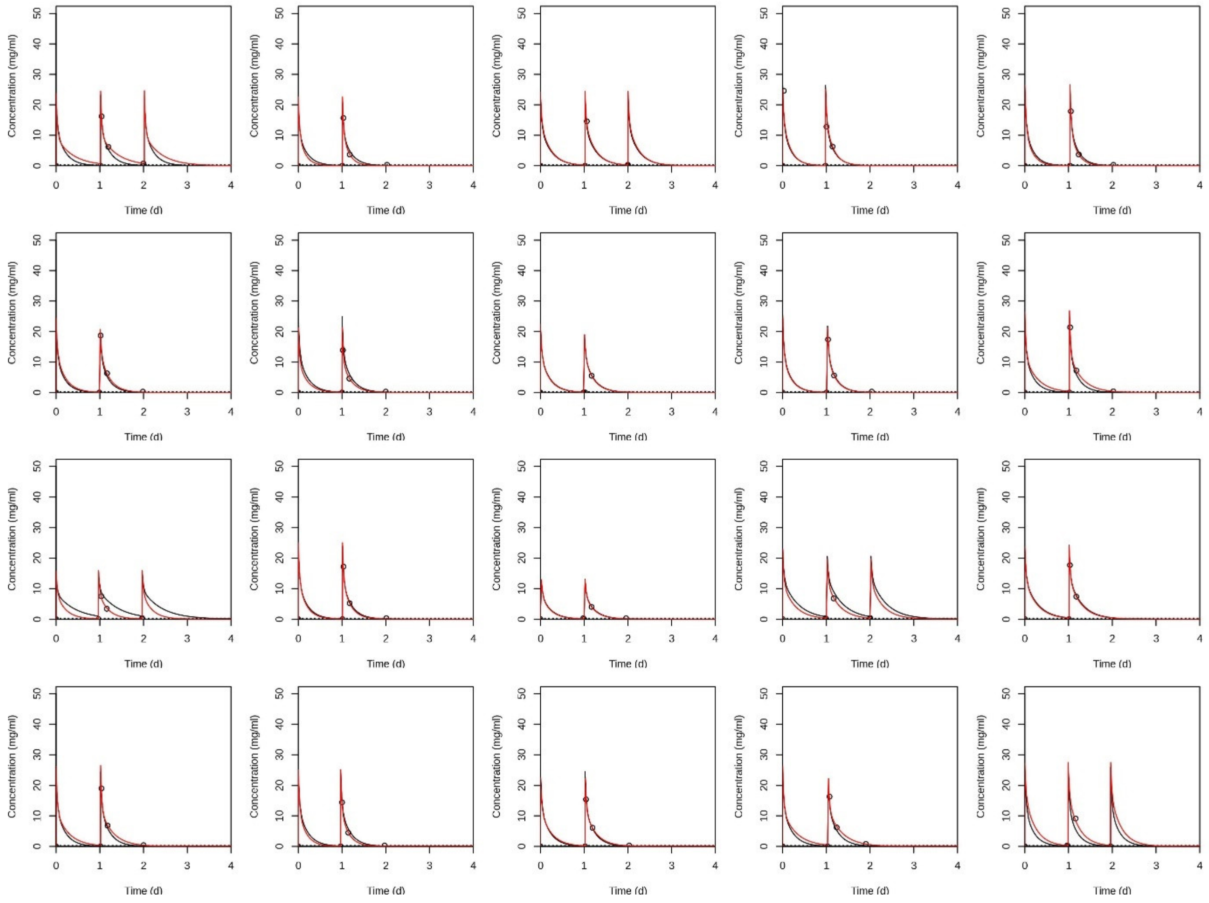
Concentration (mg/ml)
Time (d)

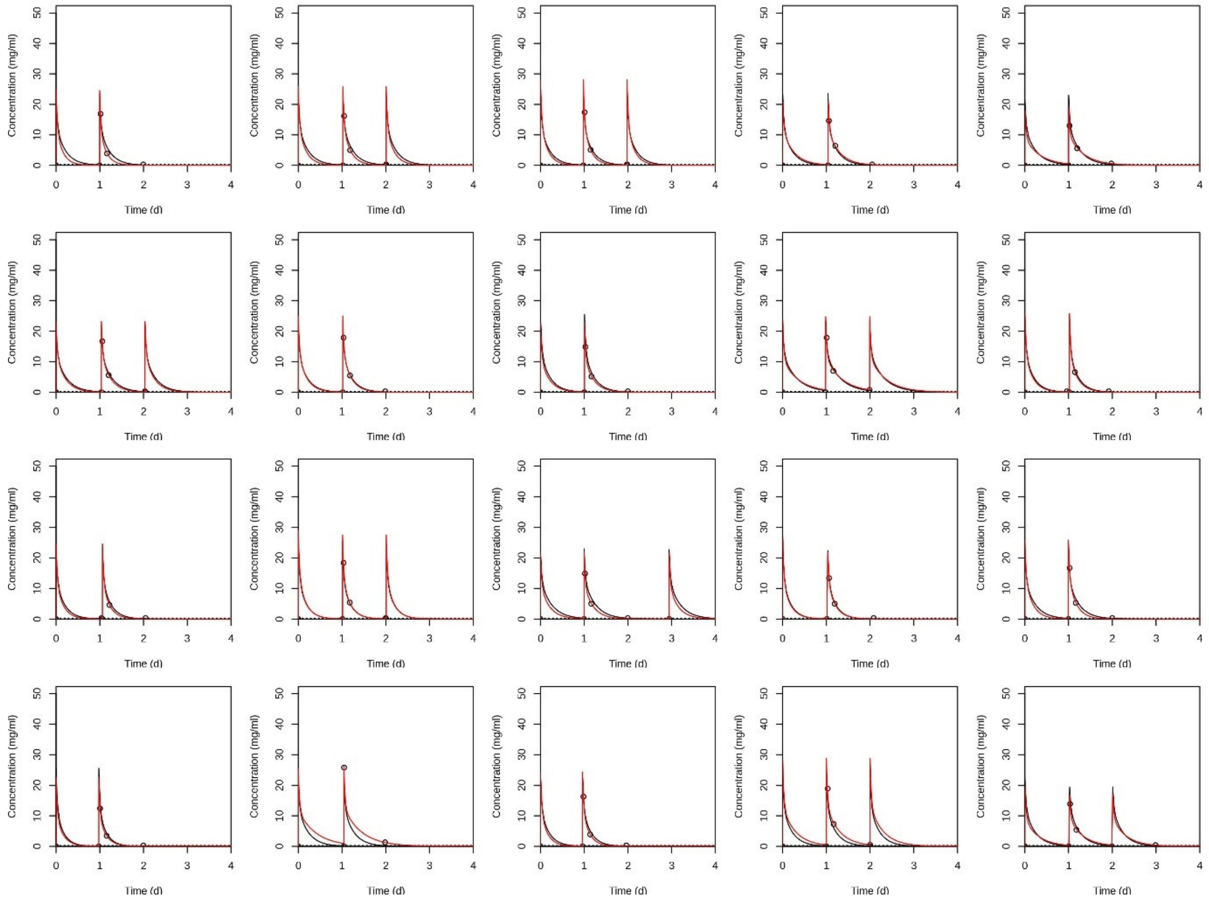
Concentration (mg/ml)
Time (d)

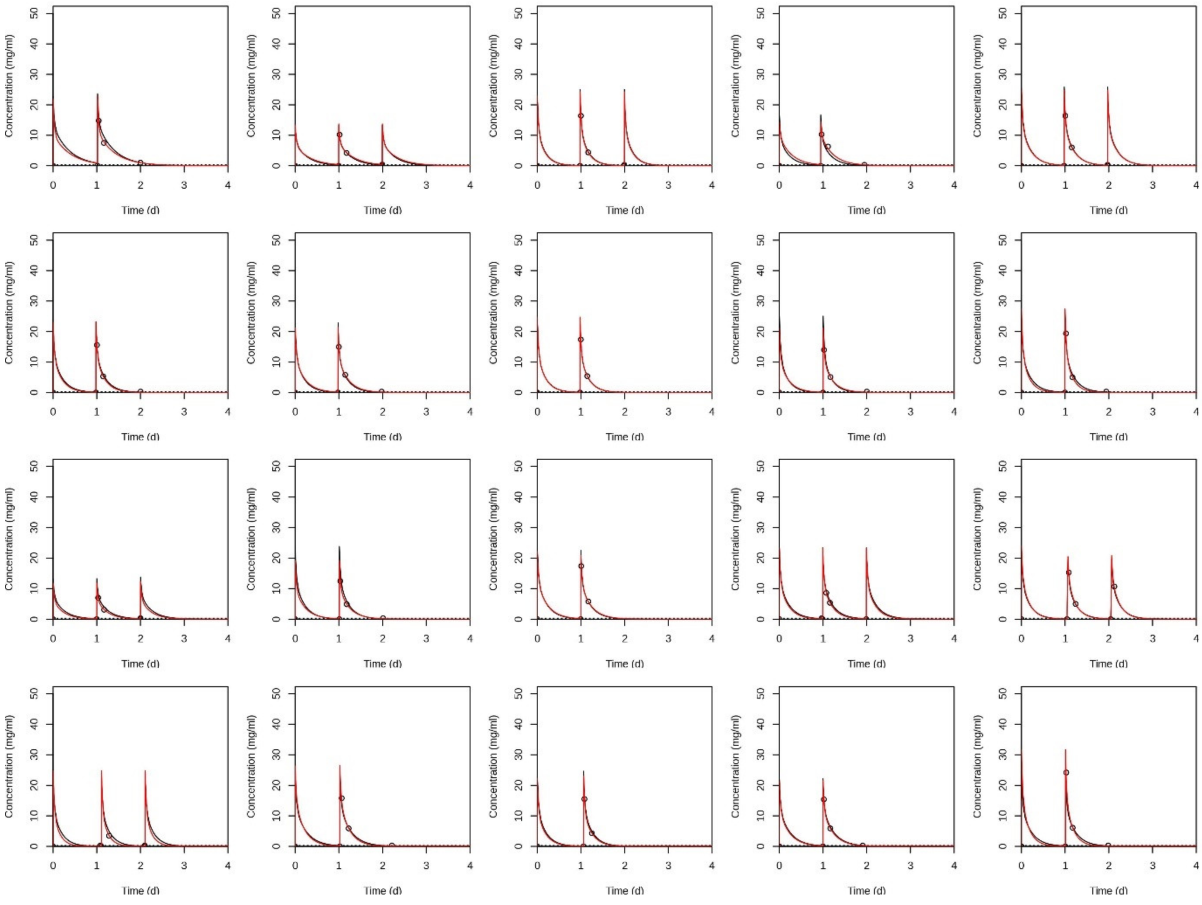
Concentration (mg/ml)
Time (d)

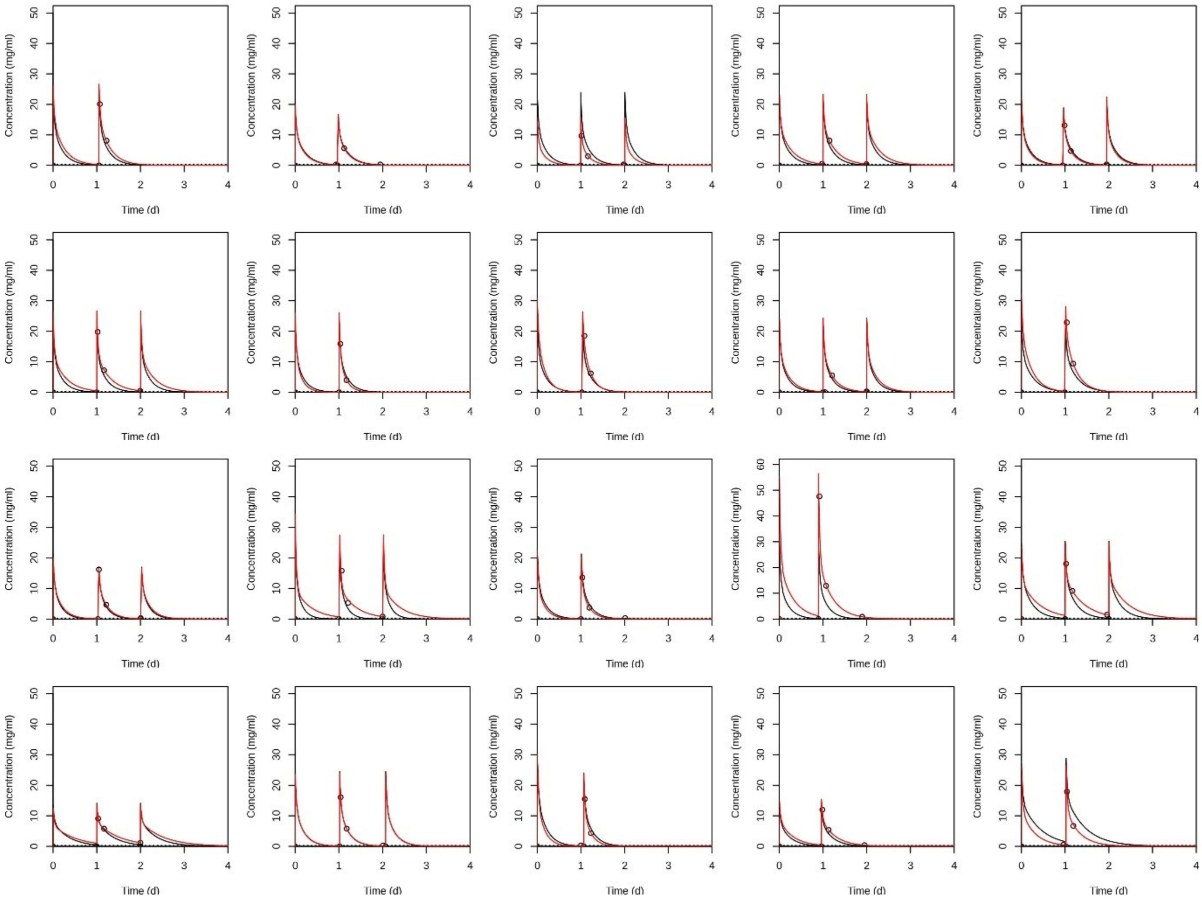
Concentration (mg/ml)
Time (d)

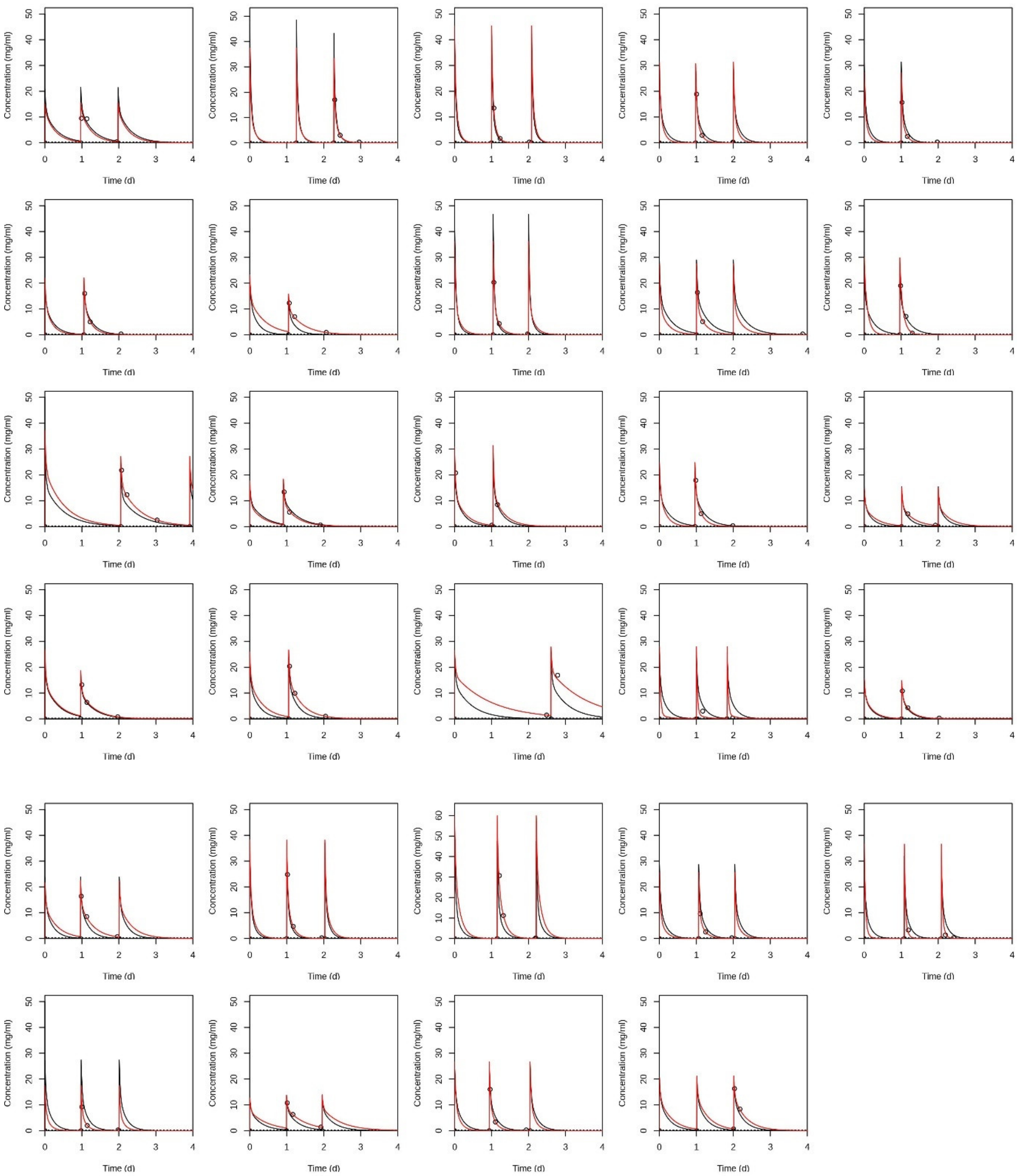

Figure S12. Measured plasma concentrations (circles) and those simulated with the final model (solid lines) plotted against time. Black solid lines, population level. Red solid lines, individual level. Black broken horizontal line, LOQ.

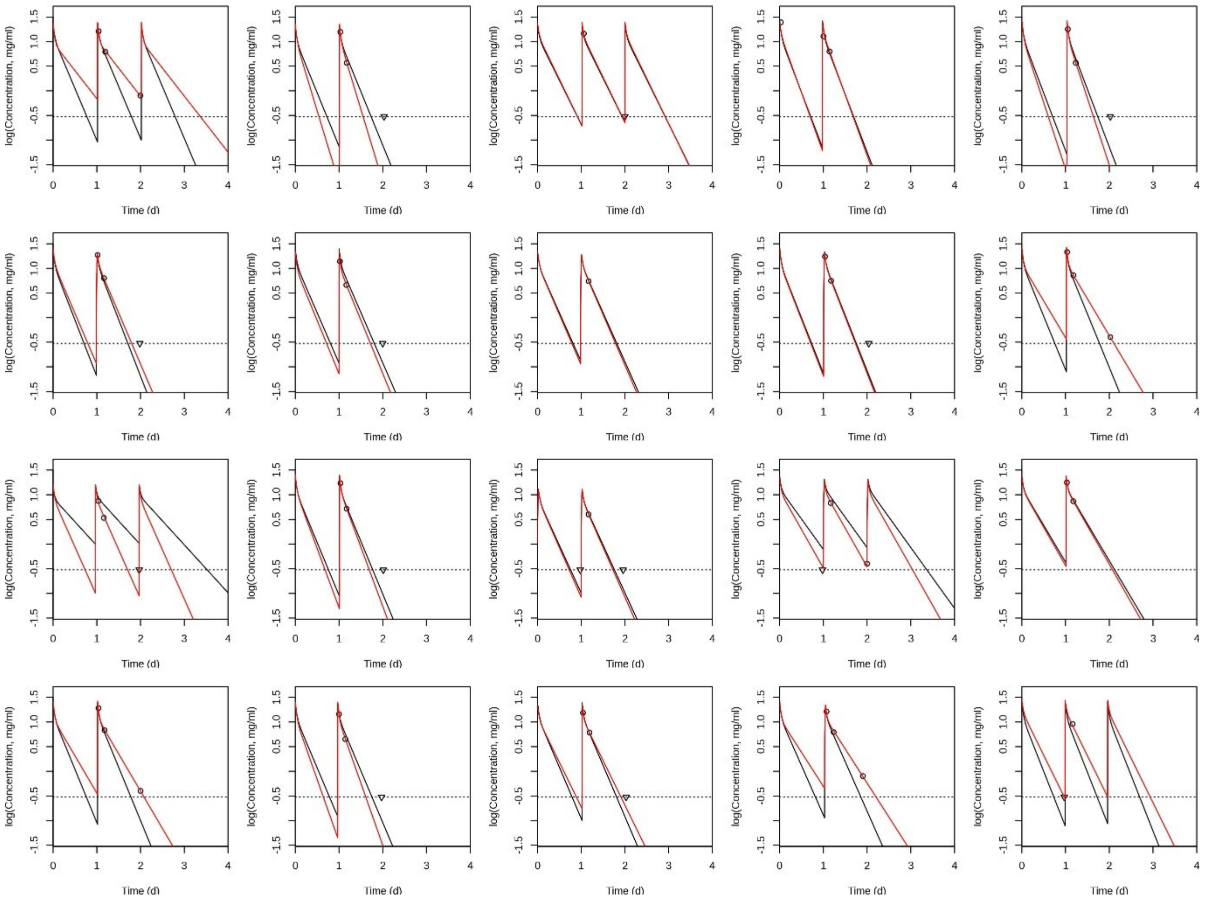
log(Concentration, mg/ml)
Time (d)

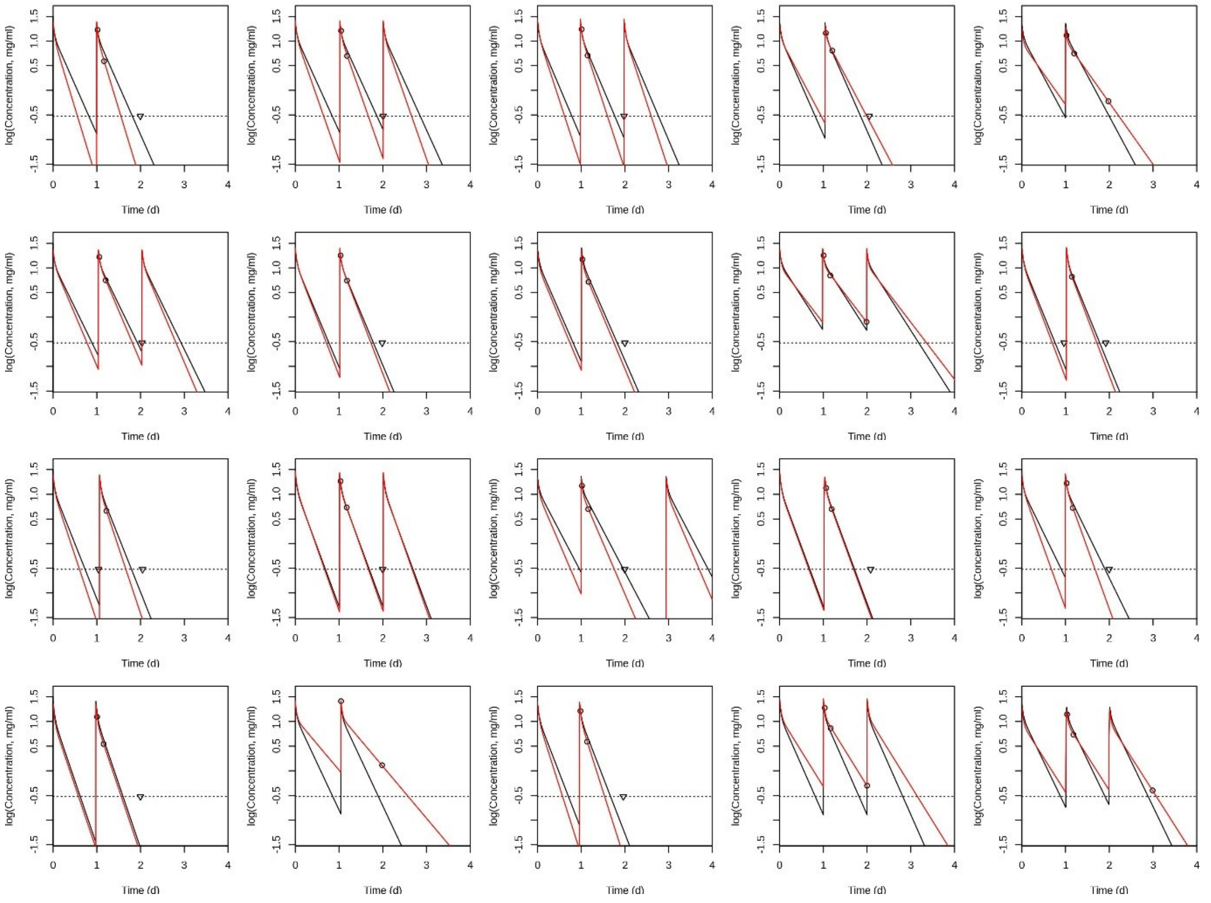
log(Concentration, mg/ml)
Time (d)

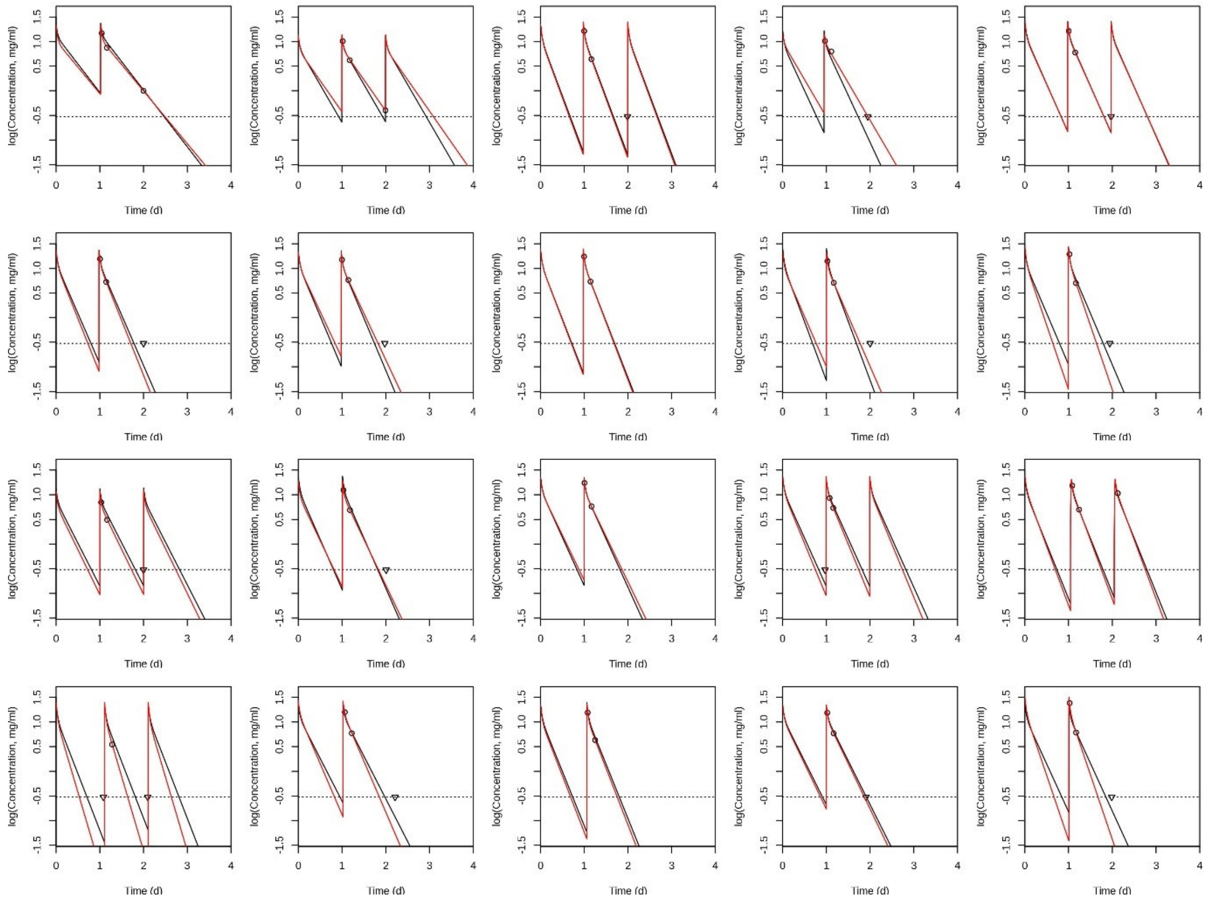
log(Concentration, mg/ml)
Time (d)
-1.5 -0.5 0.5 1.0 1.5
0 1 2 3 4

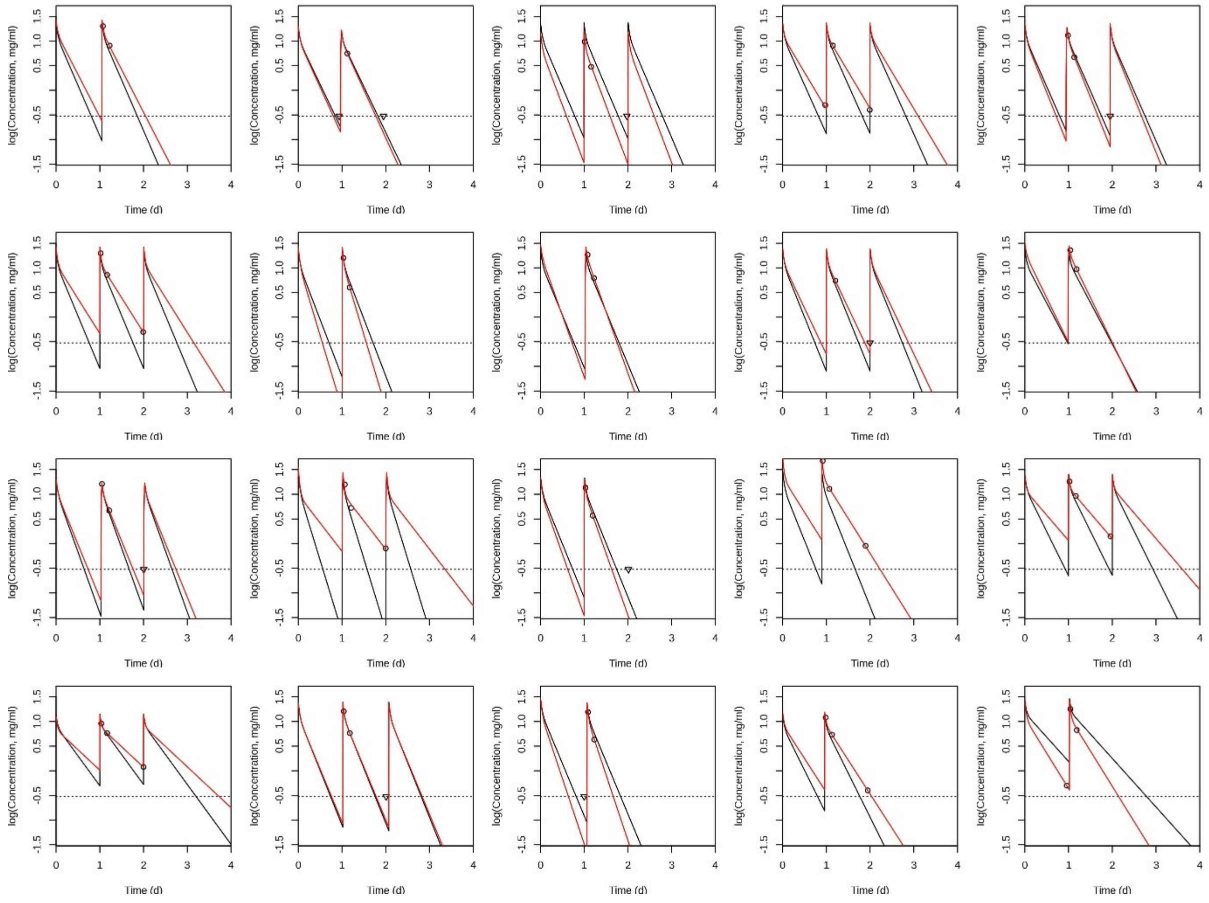
log(Concentration, mg/ml)
Time (d)
-1.5 -0.5 0.5 1.0 1.5
0 1 2 3 4

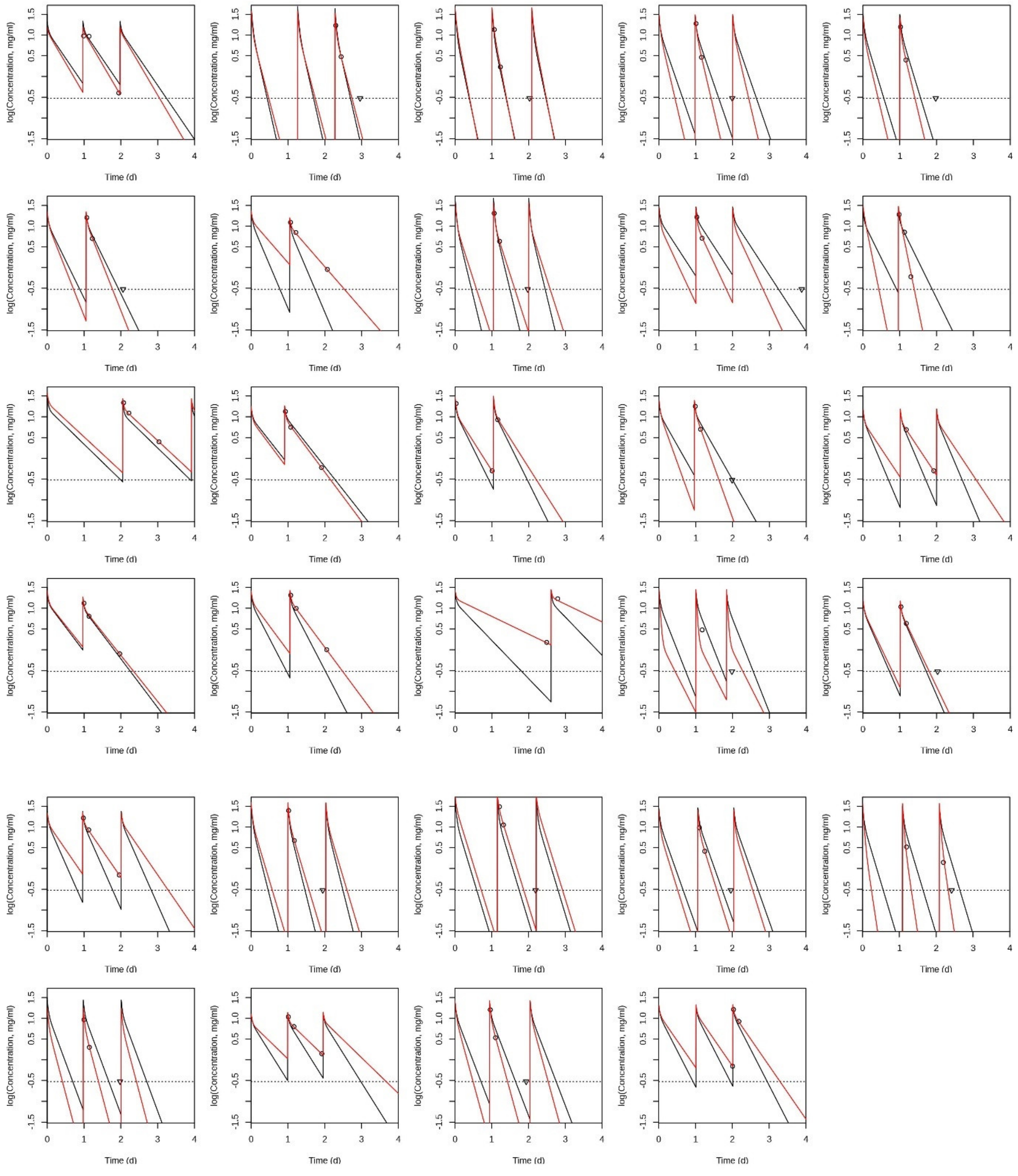

Figure S13. Measured plasma concentrations (circles) and those simulated with the final model (solid lines) plotted against time, concentrations in logarithmic scale. Black solid lines, population level. Red solid lines, individual level. Triangles, values below LOQ (0.3 mg/l), indicated at LOQ (dotted line).

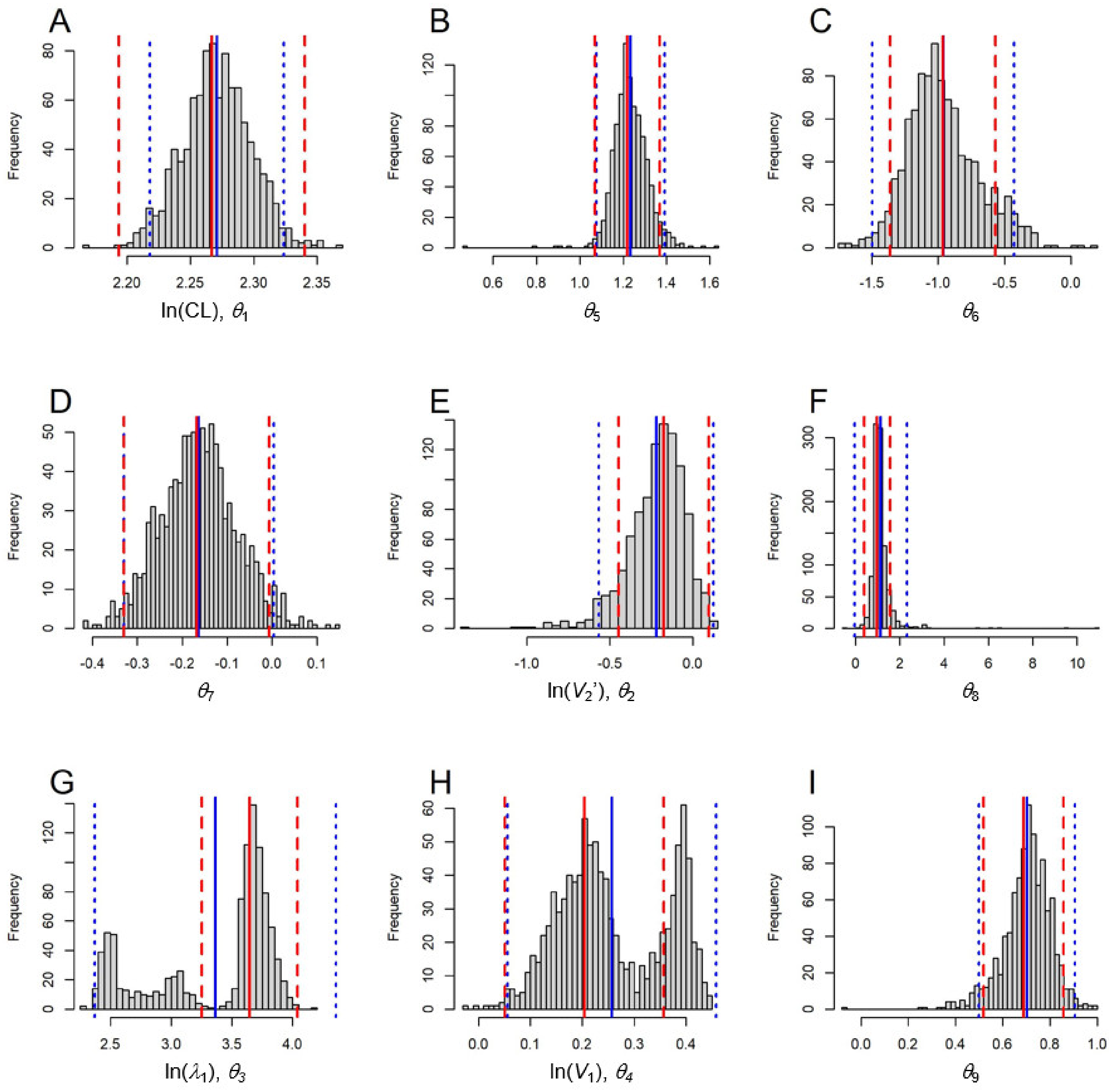

Figure S14. Fit parameters from bootstrapping. Histograms, results from the generated individual 1000 re-sampled data sets. Blue vertical lines, mean value of the 1000 bootstrapping results (solid line) and the respective 95% calculated confidence interval (assuming normal distribution; dotted lines). Red vertical lines, mean (solid line) and 95% calculated confidence interval (assuming normal distribution; broken lines) from the fit with the complete data set (values shown in Table 3). **A**) θ_1, ln(CL) intercept; **B**) θ_5, factor for ln(CL) covariate [ln(weight)-ln(4kg)]; **C**) θ_6, factor for ln(CL) covariate [ln(creatinine)-ln(27µM)]; **D**) θ_7, factor for ln(CL) covariate [ln(urea)-ln(3mM)]; **E**) θ_2, ln(V_2') intercept; **F**) θ_8, factor for ln(V_2') covariate [ln(weight)-ln(4kg)] ; **G**) θ_3, ln(λ_1) intercept; **H**) θ_4, ln(V_1) intercept; **I**) θ_9, factor for ln(V_1) covariate [ln(weight)-ln(4kg)].

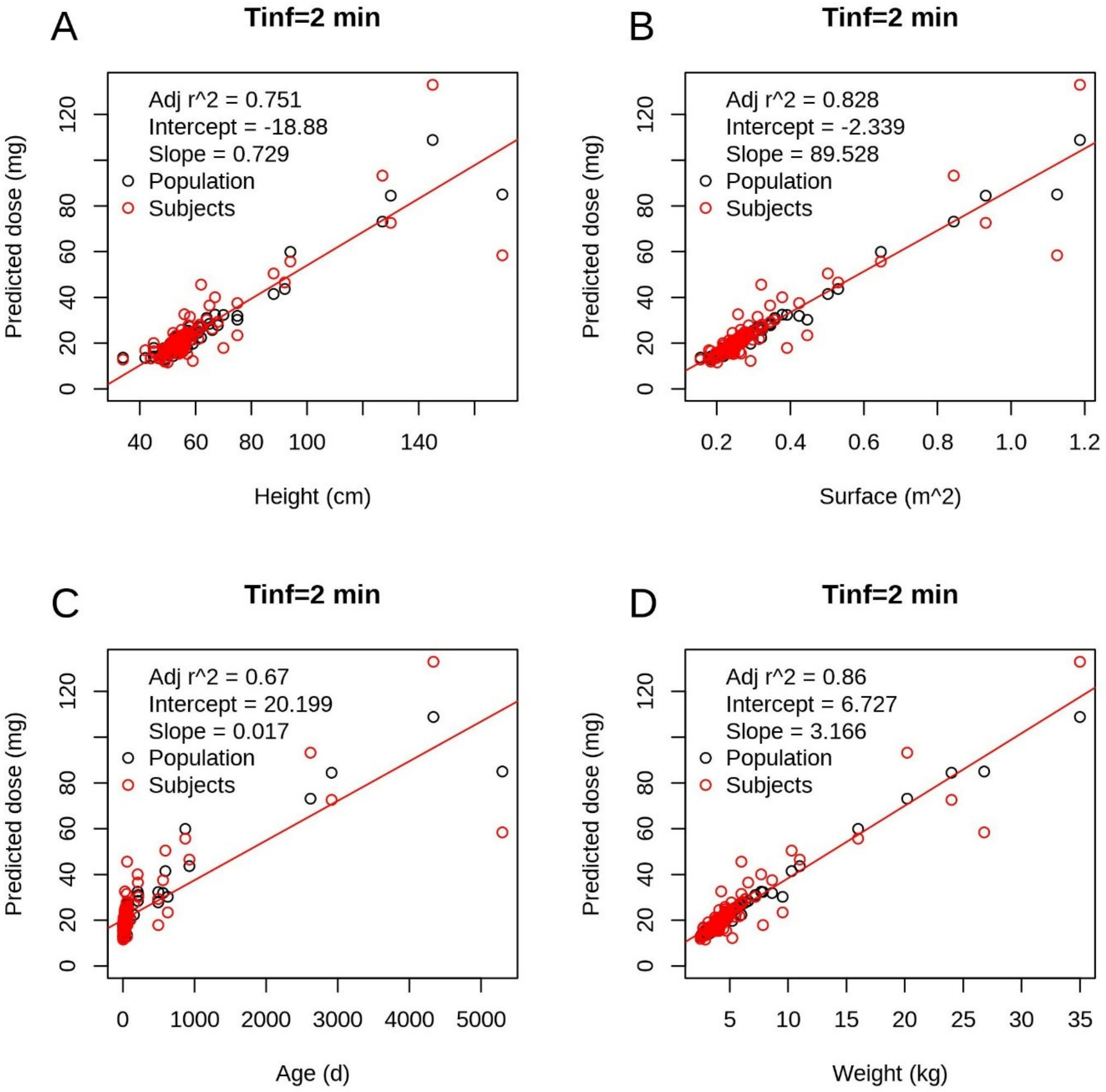

Figure S15. Predicted doses to reach *C*(30min') between 10 and 12 mg/l, calculated with the final model for a 2 min infusion. Comparison of the predicted dose with **A**) patient height, **B**) body surface area, **C**) age and **D**) body weight. Black symbols, population level; red symbols, individual level. Red lines, linear regression (predicted dose individual level *vs* patient characteristic), parameters indicated in the Figure (intercept in mg, slope in mg/kg).

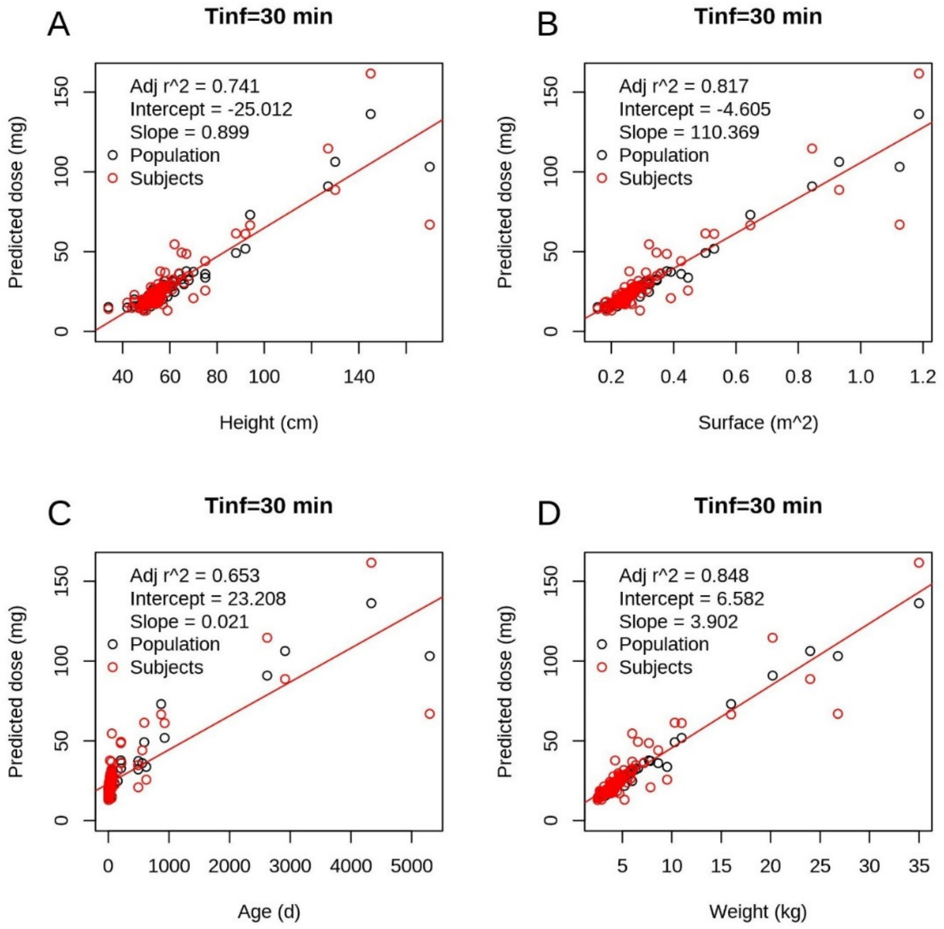

Figure S16. Predicted doses to reach *C*(30min') between 10 and 12 mg/l, calculated with the final model for a 30 min infusion. Comparison of the predicted dose with **A**) patient height, **B**) body surface area, **C**) age and **D**) body weight. Black symbols, population level; red symbols, subject level. Red lines, linear regression (predicted dose individual level *vs* patient characteristic), parameters indicated in the Figure (intercept in mg, slope in mg/kg).

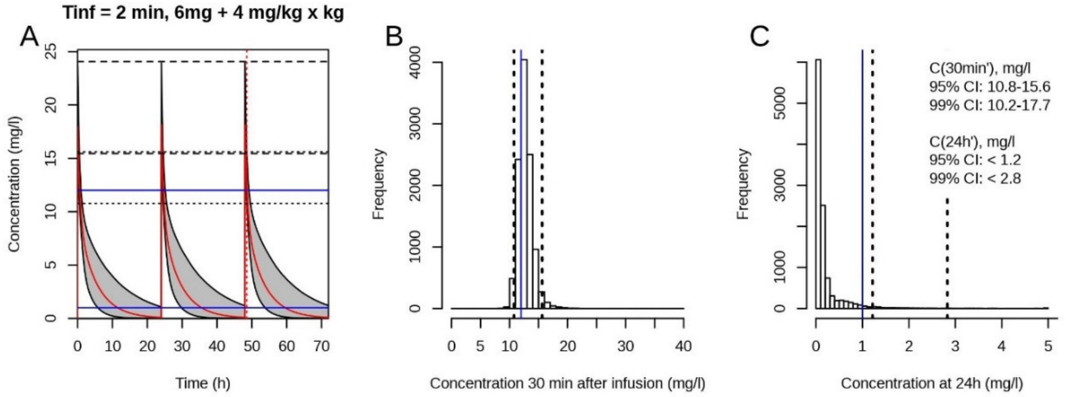

Figure S17. Plasma concentrations simulated with the final model to reach *C*(30min') between 10 and 12 mg/l. Simulations for 2 min infusion time. **A**) Plasma-concentration time curves. Grey, 95% confidence interval of the simulated concentrations (from 100 simulations per subject, *i.e.*, 10,900 simulations in total). Red, median *C*(*t*) at each time point. Blue horizontal lines, recommended maximal trough (1 mg/l), and maximal *C*(30min') level for long duration treatment (12 mg/l). Horizontal dotted and broken lines, 95% confidence intervals of the simulated *C*(30min') and maximal levels (at infusion stop), respectively. Red vertical dotted line, time point of *C*(30min'). **B**) Distribution of the simulated *C*(30min'). Vertical blue line, 12 mg/l. Vertical black broken lines, 95% confidence interval of the simulated *C*(30min'). **C**) Distribution of the simulated *C*(24h'). Vertical blue line, 1 mg/l. Vertical black broken lines, 95% and 99% upper confidence intervals of the simulated *C*(24h'). The 95% and 99% confidence interval values for the simulated *C*(30min') and *C*(24h') are provided in **C**.

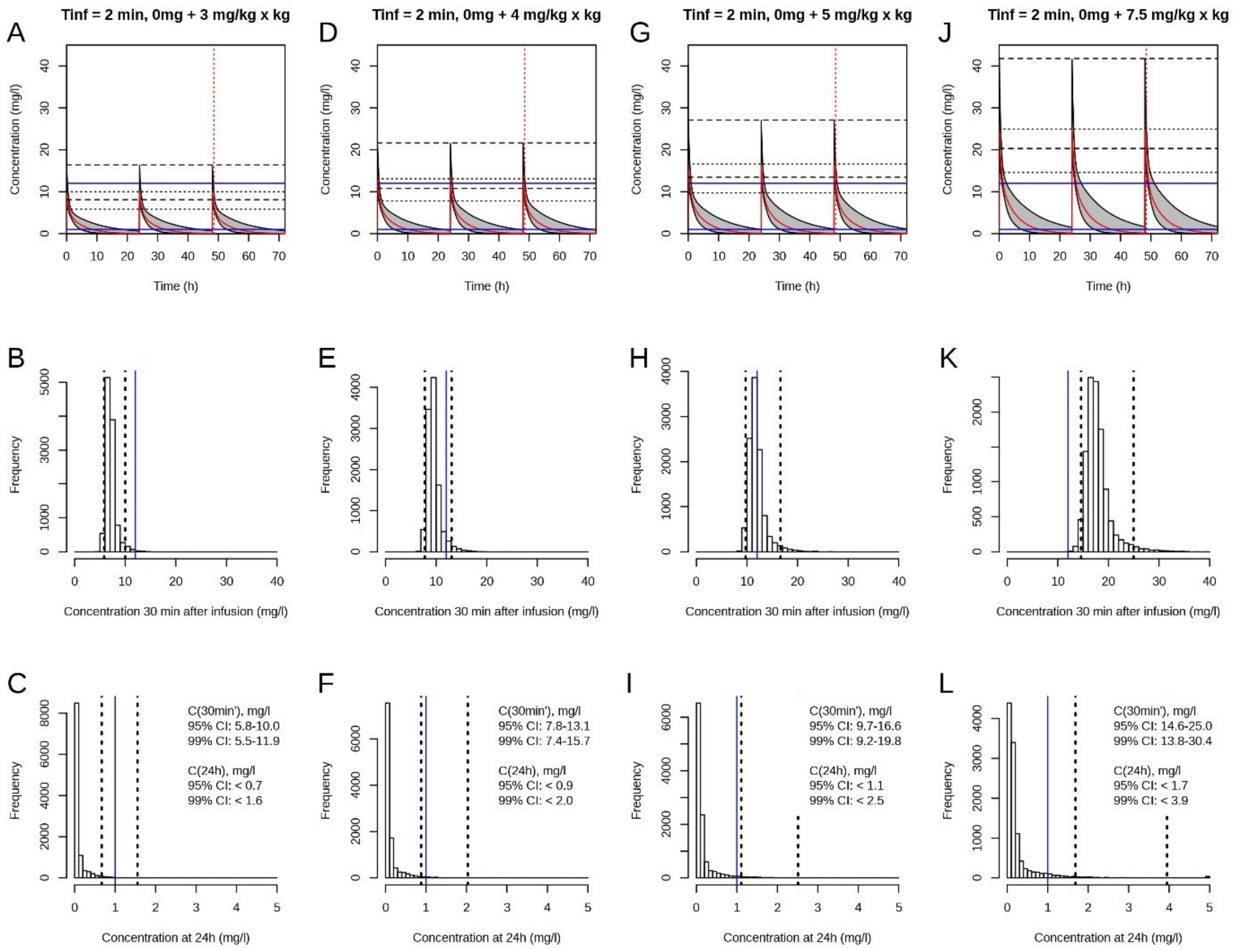

Figure S18. Simulations with the final model over 3 dosing intervals of 24 h each with 2 min infusion time. Once daily dose was **A**-**C**) 3 mg/kg; **D**-**F**) 4 mg/kg; **G**-**I**) 5 mg/kg; **J**-**L**) 7.5 mg/kg. **A**,**D**,**G**,**J**) Simulated plasma-concentration time curves. **B**,**E**,**H**,**K**) Respective distributions of simulated *C*(30min') of the third infusion. **C**,**F**,**I**,**L**) Distributions of the simulated C(24h') of the third infusion. Further details, see Figure S17.

Table S1. Ranges for the generation of random start parameters. Random start parameters (natural logarithm of the PK parameters) were generated with the R function *rand* from the package *pracma* (v2.3.3).

Fit parameter	Lower limit / upper limit	Corresponding PK parameter	Lower limit / upper limit [1]
ln(CL, l/d)	-1.3 / 5.7	CL (ml/min)	0.19 / 208
ln(V_2', l)	-2.1 / 4.9	V_2' (l)	0.12 / 134
ln(λ_1, 1/d)	-0.7 / 6.3	λ_1 (1/d)	0.50 / 545
ln(V_1, l)	-2.1 / 4.9	V_1 (l)	0.12 / 134

[1] Lower and upper limits were chosen according to the references cited in the Discussion of the main manuscript. The chosen lower limit was below published values for the respective PK parameter, the chosen upper limit above.

Reference

1. Schwartz, G.J.; Furth, S.L. Glomerular filtration rate measurement and estimation in chronic kidney disease. *Pediatr Nephrol* **2007**, *22*, 1839-1848, doi:10.1007/s00467-006-0358-1.
